# Socioeconomic Factors Affect Disparities in Access to Liver Transplant for Hepatocellular Cancer

**DOI:** 10.1155/2012/870659

**Published:** 2012-12-04

**Authors:** Linda L. Wong, Brenda Y. Hernandez, Cheryl L. Albright

**Affiliations:** ^1^Transplant Center, The Queen's Medical Center, Honolulu, HI 96813, USA; ^2^Cancer Center, University of Hawaii, Honolulu, HI 96813, USA; ^3^School of Nursing and Dental Hygiene, University of Hawaii, Honolulu, HI 96813, USA

## Abstract

*Objective*. The incidence/death rate of hepatocellular cancer (HCC) is increasing in America, and it is unclear if access to care contributes to this increase. *Design/Patients*. 575 HCC cases were reviewed for demographics, education, and tumor size. *Main Outcome Measures*. Endpoints to determine access to HCC care included whether an eligible patient underwent liver transplantation. *Results*. Transplant patients versus those not transplanted were younger (55.7 versus 61.8 yrs, *P* < 0.001), males (89.3% versus 74.4%, *P* = 0.013), and having completed high school (10.1% versus 1.2%, *P* = 0.016). There were differences in transplant by ethnicity, insurance, and occupation. Transplant patients with HCC had higher median income via census classification ($54,383 versus $49,383, *P* = 0.046) and self-reported income ($48,948 versus $38,800, *P* = 0.002). Differences in access may be related to exclusion criteria for liver transplant, as Pacific Islanders were more likely to have tumor size larger than 5 cm compared to Whites and have BMI > 35 (20.7%) compared to Whites (6.4%) and Asians (4.7%). *Conclusions*. Ethnic differences in access to transplant are associated with socioeconomic status and factors that can disqualify patients (advanced disease/morbid obesity). Efforts to overcome educational barriers and screening for HCC could improve access to transplant.

## 1. Introduction

Hepatocellular cancer is the fifth most common cancer worldwide and is the fifth leading cause of cancer death in males in the USA. Although cancer incidence in the USA is generally decreasing, HCC is one of a few cancers that is increasing in incidence and death rate [1, 2]. The best treatment for long-term disease-free survival with hepatocellular cancer is liver transplantation. Those who qualify for liver transplant must have localized disease, not amenable to surgical resection, and access to donor livers. Because of limited donor livers, criteria have been developed to transplant those patients with HCC who have the best prognosis. The recommended criteria include tumor characteristics—specifically Milan criteria with a single tumor less than 5 cm or 3 tumors all less than 3 cm and without evidence of vascular invasion or extrahepatic spread of tumor [3, 4]. There are also other criteria for liver transplant that relate to the presence of other medical comorbidities, psychosocial factors, and the ability to finance the transplant procedure. Some of the latter criteria vary from center to center, but all centers aim to transplant those patients who are a reasonable operative risk and who demonstrate adequate compliance, psychosocial support, and financial means to deal with the immunosuppressive regimens and possible side effects of transplant.

Overall, HCC is being identified at earlier stages, and survival is improving as more cases are diagnosed and treated at early stages [5]. However, disparities in liver transplant for all causes and disparities in treatment for HCC have been reported. Specifically, females have been reported to be disadvantaged in terms of overall liver transplant rates [6]. African Americans and Asian/Pacific Islanders are less likely to receive a liver transplant for HCC than White patients [7]. Differences in access to liver transplant have also been reported depending on insurance status, geography, and income status [8–10]. The purpose of this study is to delve more deeply into the reason for disparities in access to liver transplant for patients with HCC. In particular we wanted to determine if there were certain factors that were important in these disparities, including educational background, occupation, insurance status, and proximity to the transplant center.

## 2. Methods and Materials

### 2.1. Patients

This is a retrospective analysis of 749 HCC cases referred to a group of surgeons (LW) who specialize in hepatobiliary surgery. This group is affiliated with Hawaii's only clinic dedicated to liver diseases, the only liver transplant center in the State and the only referral center for liver diseases and surgery for American territories of the Pacific Basin (including American Samoa, Guam, Saipan, and the Marshall Islands). In addition, a number of patients were foreign nationals from Asian countries, including China, Japan, Korea, and the Philippines, who sought medical care in the USA. These surgeons see about 60%–70% of the HCC cases in the State of Hawaii.

HCC was diagnosed histologically by percutaneous biopsy, liver biopsy at the time of surgery, or examination of the resected liver. Before 2010, according to the United Network for Organ Sharing policy regarding transplant for HCC, patients without histologic confirmation were included if they had a history of chronic liver disease and a mass at least 2 cm in size seen on two imaging studies (ultrasound, CT scan, or MRI) and one of the following: (1) a vascular blush seen on CT scan or MRI, (2) Alpha fetoprotein (AFP) > 200 ng/mL, or (3) an arteriogram confirming the tumor. Since 2010 and consistent with the American Association for the Study of Liver Disease (AASLD) guidelines, patients without histologic confirmation were included if they had a contrast enhanced study that demonstrated a tumor larger than 1 cm with hypervascularity in the arterial phase and washout in the portal vein or delayed phase. If the findings were not typical, then a second contrast-enhanced study or biopsy was used to confirm the diagnosis [4, 11].

Information on demographics, medical history, laboratory results, tumor characteristics, treatment, and survival data was collected via a clinical interview. Demographic data included age, sex, birthplace, and the patient's self-reported ethnicity. Ethnicity was categorized as “White,” “Asian” (including Filipinos), or “Pacific Islander.” Patients who did not fit into one of these categories or were of mixed ethnicity were subsequently classified as “Other.” Patients of mixed race with 50% Pacific Islander ethnicity were categorized as “Pacific Islander.” Measured height and weight were used to determine body mass index (BMI). BMI ≥ 35 was a relative contraindication to liver transplants at this center.

Patients were asked about the years of education that they completed. Those that reported that they successfully completed the General Educational Development test (GED) were recorded as having finished high school or 13 years of education. We did not have access to information as to whether English was the primary language spoken; however Hawaii has about 25.5% of households in which English is not the primary language spoken compared to 20.1% for the remainder of the USA [12].

Patients were also asked about their current occupation. This was noted in detail and later categorized into “White Collar” including professional, semiprofessional, administrative, and salaried workers, “Blue Collar” denoting those who perform manual labor, “Service workers,” and “None”. “None” also included those patients who were on disability, retired, homemakers, unemployed but looking for work and those who had never been employed. Cases in which there was no information on years of education or current occupation were eliminated from this study; thus only 575 cases of the 749 were included.

Insurance status was categorized as “Medicare,” “Private” (includes health maintenance organizations), “Medicaid” (any type of state government assisted programs), or “None.” Ten patients were referred from the Veterans Administration (VA) solely for liver transplant evaluation and were not included in the analysis of insurance status. All other treatments for HCC within the VA system were conducted by their center's staff oncologists and surgeons.

Each patient had zip code of residence noted, and based on this information, they were categorized into “Oahu” versus “Non-Oahu” as a surrogate for urban versus rural. The island of Oahu includes Honolulu, the State's capital and largest metropolitan city and the location of the only dedicated liver transplant and treatment facility. HCC patients from other islands are typically required to travel to Honolulu for evaluation.

Median income was determined with two different methods. The first was based on zip code using the US Census data 2010 [13]. Median income was not available in 48 patients with missing residential addresses or unavailable zip code income data.The second method used highest level of education attained to estimate income based on 2010 US income by education data [14].

### 2.2. Treatments

Treatments included liver resection, transplantation, systemic chemotherapy, and ablative therapies (including radiofrequency ablation, cryosurgery, transarterial chemoembolization, and percutaneous ethanol injection). Liver resection was considered in Child's A patients and early Child's B patients (Child's Turcotte-Pugh score of 7, without any evidence of ascites or encephalopathy). Liver transplants were considered in patients who were unresectable but met Milan criteria (single tumor less than 5 cm or 2 to 3 tumors, each less than 3 cm). Liver transplant was also performed in patients who underwent resection but had a recurrence more than six months after surgery, provided the recurrent tumor met Milan criteria, and there was no disease progression while awaiting transplant. Since 2007, liver transplant was considered in single tumors less than 6.5 cm that were downstaged to meet Milan criteria. All liver resections and transplants were performed by members of our surgical group. The majority of patients on the transplant list underwent either percutaneous radiofrequency ablation or transarterial chemoembolization while waiting for a donor.

### 2.3. Data Endpoints

Access to health care was measured by whether a patient underwent liver transplantation. These groups were analyzed by age, gender, ethnicity, education (finished high school versus did not finish high school), median income, and insurance status.

These groups were analyzed in more detail in terms of factors which might affect access to transplant including morbid obesity, tumor size > 5 cm, and tumor outside Milan criteria which were contraindication at this transplant center.

### 2.4. Data Analysis

All analyses were performed using SPSS statistical software. From the database of 749 patients, a total of 575 were retained for the present analysis. Comparisons for transplant versus no liver transplant (in patients clinically eligible for transplant) were compared using the chi square test. Factors associated with and transplant were evaluated using unconditional logistic regression to calculate crude and age-adjusted odds ratios (OR) and 95% confidence intervals (CI).

## 3. Results

A total of 575 HCC patients were evaluated including 436 males and 139 females, with mean age being 61.2 years. Racial distribution was as follows: Asian—350, White—119, Pacific Islander—86, Mixed (more than 2 races)—7, Hispanic—6, Black—4, and other—3. The 20 patients identified as Mixed, Hispanic, Black, and other were excluded from analysis of race to small numbers. Birthplace was primarily in the USA (341 patients), but 195 were born in an Asian country, 27 were born in a Pacific Island nation (or US territory), and 3 patients were born elsewhere.

Overall, most patients had some type of medical insurance, including Private insurance—300, Medicare—167, Medicaid—92, and Veterans Administration (VA) insurance—10. Only 3 patients were uninsured in this cohort. Of the 398 patients in whom educational background was recorded, 316 (79.3%) completed high school or higher education. Occupational status was known in 515 patients, and 86.8% were currently employed. Distribution of types of occupation included blue collar—222, service—147, and white collar—138. Sixty-eight patients either were disabled, retired, or currently unemployed.

Overall tumor characteristics included the following distribution by stage: I—342 patients, II—8 patients, III—139 patients, and IV—12 patients. More patients had the largest tumor 5 cm or larger (320 patients) compared to those with largest tumor less than 5 cm (241 patients). Of the 575 patients in the cohort, 258 (44.9%) met Milan criteria.

Of the 575 patients, 521 had a chronic liver disease or viral hepatitis, and 54 had no underlying disease for which screening could have been recommended or performed. Etiology of HCC varied by race with hepatitis B related HCC predominant in Asians and hepatitis C in Whites (Table 1). Eight patients had HCC found incidentally on the explanted liver at the time of transplant and were excluded from the analysis of screening versus nonscreening. Fifty-six patients (9.7%) patients underwent liver transplant.

Patients who underwent liver transplant for HCC were more likely to be younger in age and male (Table 2). Pacific Islanders were less likely to receive transplantation. Liver transplant patients were also more likely to have finished high school and have private insurance. Patients with no listed occupation (unemployed, disabled, currently not working) were less likely to receive a transplant. Location of residence did not matter. Median income as estimated by both zip code and education level was significantly higher in patients who underwent liver transplant. Patients who underwent liver transplant for HCC had higher median income based on zip code ($54,383 versus $49,383, *P* = 0.046) and based on self-reported education level ($48,948 versus $38,800, *P* = 0.002).

Factors of race, education, insurance, and occupation were then analyzed as to how they may affect the main criteria contraindicating liver transplant for HCC which include tumor size > 5 cm, BMI > 35, and outside Milan criteria (Table 3). Level of education and type of insurance did not seem to affect the presence of these criteria. In terms of race, Pacific Islanders were more likely to have tumor size larger than 5 cm when compared to Whites. Pacific Islanders also had significantly more patients (20.7%) with BMI > 35 compared to Whites (6.4%) and Asians (4.7%).

With unconditional logistic regression, the only factors that affected access to transplant include age > 60 years (*P* < 0.001) and insurance status (*P* = 0.001).

## 4. Discussion

It is not a great surprise that there are disparities in access to liver transplant for HCC. With the limited donor livers, expense, and the need for criteria to select patients, herein lies the problem of access to liver transplant. Identifying tumors that meet Milan criteria can be difficult as HCC is generally not symptomatic until advanced stages of liver disease or cancer are present. The best way to identify tumors at earlier stage would be to identify the population at risk—those with viral hepatitis or some type of cirrhosis—and screen them for HCC. Thus access to liver transplant for HCC is intimately related to a patient's access to health care and screening for HCC.

Studies have demonstrated ethnic and gender disparities in access to liver transplant. Mathur et al. in reviewing the Scientific Registry of Transplant Recipients (SRTR) data (*n* = 79, 998) showed that females had a lower transplant rate in both the pre-MELD (9%, *P* < 0.0001) and MELD eras (14%, *P* < 0.0001) and that there was substantial geographic variation in these sex-based differences [15]. In a separate study, this group determined that African Americans and Asians had similar liver transplant rates compared to Whites, but Hispanics had an 8% lower transplant rate compared to Whites, and subgroups of Asian candidates with higher MELD scores had a 46% lower transplant rate [6, 16]. Differences in transplant may also be due to timing of referral to a transplant center as several studies have demonstrated that African Americans and ethnic minorities had delayed referral or higher MELD score at the time of referral [17–19]. Other factors affecting transplant access include insurance status and distance from the transplant center [20–22].

Studies have also demonstrated differences in access to care and survival in patients with HCC. In reviewing the California Cancer Registry with 12,148 HCC cases, African American and Hispanic patients were less likely than White patients to undergo liver transplant for HCC, and those with lower socioeconomic status and no private insurance were less likely to receive surgery of any type. Those in the highest socioeconomic quintile were four times more likely to receive a liver transplant than the lowest quintile [23]. Mathur et al. in reviewing the SEER HCC data showed that there were still racial disparities in survival even when considering early stage HCC. Blacks had persistently poor survival even after accounting for differences in stage and use of invasive therapy [24]. The SEER data also showed that Asians/Pacific Islanders had better 5-year survival (23%) when compared to Whites (18%), Hispanics (15%), or Blacks (12%) [5].

Our study demonstrates that there are differences in access to liver transplant by age, gender, ethnicity, insurance, level of education, median income, and occupation, but with the multivariate analysis, insurance status and age appeared to be the most important. Proximity to a transplant center that requires travel to another island did not seem to affect access. In particular, the Pacific Islanders underwent fewer liver transplants compared to Whites and Asians. In trying to determine factors that may account for this, we demonstrate that fewer Pacific Islanders had their HCC found on screening, and more of them had tumors larger than 5 cm which may have disqualified them; however the same proportion of their HCC did meet Milan criteria. Morbid obesity (BMI > 35) is a relative contraindication to liver transplant at our center, and more Pacific Islanders had BMI > 35 compared to Whites and Asians, which may have contributed to the fewer transplants performed in this ethnic group. Since high BMI has been associated with lower socioeconomic status, our relative contraindication of BMI > 35 may have contributed to the low transplant rate in those with lower socioeconomic status. However with multinomial regression, the most important factors in determining transplant access included age and insurance status. The most important determinant of screening was ethnicity and gender.

This study is somewhat limited as it is a single center study in a primarily Asian population. However, larger studies with administrative databases such as the Medicare database and the UNOS/SRTR will not have information on patient's educational background, occupation, and proximity to transplant centers. Large single center studies or collaborations with individual centers will be necessary to truly investigate cultural and psychosocial barriers to care. In addition, “Asians” and “Pacific Islanders” are frequently combined in most US studies, when our study clearly shows that there are differences in access to HCC screening and transplant in these two groups.

It will be difficult to overcome some of the barriers to liver transplant access in HCC primarily because limited donor livers drive the need for strict criteria. Patients must have enough of an understanding of their disease to demonstrate compliance with posttransplant care which may exclude some patients with limited education. Patients must also be able to finance such an expensive therapy which likely limits the treatment to those with medical insurance, an occupation that provides medical insurance or some other financial means.

## 5. Conclusions

We have demonstrated that there are differences in socioeconomic factors such as age, gender, ethnicity, and educational background that affect whether a patient had their liver cancer found with screening or underwent transplant. We need to better educate the community on liver disease and to better identify HCC at an earlier stage with surveillance, especially in Pacific Islanders, so more patients can qualify for some type of therapy, including liver transplant.

## Figures and Tables

**Table 1:** Etiology of HCC cases^1^ by race.

	Hepatitis B	Hepatitis C	Alcohol
Overall	231/575 (40.2%)	234/575 (40.1%)	252/575 (43.8%)
Asians	165/350 (47.1%)	104/350 (29.7%)	115/350 (32.9%)
Whites	19/119 (16.0%)	82/119 (68.9%)	73/119 (61.3%)
Pacific Islanders	41/86 (47.7%)	34/86 (39.5%)	51/86 (59.3%)

^1^Excludes cases without a history of HBV, HCV, and/or excess alcohol use.

**Table 2:** HCC patients by liver transplant status.

	Transplant (*n* = 56)	No transplant (*n* = 519)	*P* value/unadjusted Odds ratio (95% CI)	Age-adjusted^1^ Odds ratio (95% CI)
Age (years)	55.7 ± 6.59	61.8 ± 11.55	*P* < 0.001	3.99 (1.90–8.35)
Sex			*P* = 0.013	
Males	50 (11.5%)	386 (88.5%)	2.87 (1.20–6.85)	2.05 (0.81–5.41)
Females	6 (4.3%)	133 (95.7%)	1.00 (Reference)	1.00 (Reference)
Race			*P* = 0.011	
Asian	32 (9.1%)	318 (90.9%)	0.53 (0.29–0.98)	0.46 (0.24–0.92)
White	19 (16%)	100 (84%)	1.00 (Reference)	1.00 (Reference)
Pacific Islander	4 (4.7%)	82 (95.3%)	0.26 (0.08–0.78)	0.35 (0.11–1.14)
Education			*P* = 0.006	
13+ years	32 (10.1%)	284 (89.9%)	9.1 (1.23–67.8)	4.24 (0.48–30.3)
<13 years	1 (1.2%)	81 (98.8%)	1.00 (Reference)	1.00 (Reference)
Insurance			*P* < 0.001	
Private	43 (14.3%)	257 (85.7%)	1.00 (Reference)	1.00 (Reference)
Medicare	10 (6%)	157 (94.0%)	0.38 (0.19–0.78)	0.75 (1.22–4.64)
Medicaid	0 (0%)	92 (100%)	—	—
Occupation			*P* = 0.017	
White collar	21 (15.2%)	117 (84.8%)	1.00 (Reference)	1.00 (Reference)
Blue collar	21 (9.5%)	201 (90.5%)	0.58 (0.31–1.11)	0.49 (0.24–1.02)
Service	13 (8.8%)	134 (91.2%)	0.54 (0.26–1.13)	0.47 (0.21–1.05)
None	1 (1.5%)	67 (98.5%)	0.08 (0.01–0.63)	5*E* − 9(5*E* − 9–5*E* − 9)
Residence			NS (*P* = 0.490)	
Oahu	43 (9.7%)	519 (90.3%)	0.81 (0.43–1.55)	0.69 (0.32–1.48)
Non-Oahu	13 (9.3%)	127 (90.7%)	1.00 (Reference)	1.00 (Reference)
Median income (based on zip code)				
<$48,264	24 (42.9%)	277 (53.4%)	1.00 (Reference)	1.00 (Reference)
≥$48,264	32 (57.1%)	242 (46.6%)	1.53 (0.88–2.66)	1.64 (0.93–2.88)
Median income (based on education)				
<$48,264	40 (71.4%)	446 (85.9%)	1.00 (Reference)	1.00 (Reference)
≥$48,264	16 (28.6%)	73 (14.1%)	2.44 (1.30–4.59)	3.04 (1.57–5.86)

**Table 3:** Differences in exclusion criteria for transplant by race, education, insurance, and occupation.

	Tumor size > 5 cm	BMI > 35	Outside Milan criteria
Race			
Asian (As)	156/34 (45.9%)	15/318 (4.7%)	199/349 (57.0%)
White (Wh)	35/116 (30.2%)	7/109 (6.4%)	59/119 (47.9%)
Pacific Islander (PI)	46/86 (53.5%)	16/77 (20.7%)	53/86 (61.6%)
	*P* = 0.001 (PI versus Wh)	*P* < 0.001 (PI versus As)	NS
		*P* = 0.0057 (PI versus Wh)	
Education			
13+ years	122/309 (39.4%)	21/305 (6.9%)	165/315 (52.4%)
<13 years	40/80 (50%)	10/77 (13.0%)	49/82 (59.8%)
	NS	NS	NS
Insurance			
Private	130/291 (44.7%)	20/281 (7.1%)	174/299 (58.2%)
Medicare	69/164 (42.1%)	8/144 (5.6%)	87/167 (52.1%)
Medicaid	36/80 (45.0%)	8/8 (9.9%)	49/92 (53.3%)
	NS	NS	NS
Occupation			
White collar (WC)	60/133 (45.1%)	10/120 (8.3%)	82/138 (59.4%)
Blue collar (BC)	97/217 (44.7%)	13/199 (6.5%)	122/221 (55.2%)
Service	64/144 (44.4%)	12/130 (9.2%)	78/147 (53.1%)
None	20/67 (29.9%)	3/6 (4.9%)	34/68 (50%)
	*P* = 0.05 (service versus WC)	NS	NS
